# Reconstruction and flux analysis of coupling between metabolic pathways of astrocytes and neurons: application to cerebral hypoxia

**DOI:** 10.1186/1742-4682-4-48

**Published:** 2007-12-10

**Authors:** Tunahan Çakιr, Selma Alsan, Hale Saybaşιlι, Ata Akιn, Kutlu Ö Ülgen

**Affiliations:** 1 Department of Chemical Engineering, Boğaziçi University, 34342, Bebek. Istanbul, Turkey; 2 Institute of Biomedical Engineering, Boğaziçi University, 34342, Bebek. Istanbul, Turkey

## Abstract

**Background:**

It is a daunting task to identify all the metabolic pathways of brain energy metabolism and develop a dynamic simulation environment that will cover a time scale ranging from seconds to hours. To simplify this task and make it more practicable, we undertook stoichiometric modeling of brain energy metabolism with the major aim of including the main interacting pathways in and between astrocytes and neurons.

**Model:**

The constructed model includes central metabolism (glycolysis, pentose phosphate pathway, TCA cycle), lipid metabolism, reactive oxygen species (ROS) detoxification, amino acid metabolism (synthesis and catabolism), the well-known glutamate-glutamine cycle, other coupling reactions between astrocytes and neurons, and neurotransmitter metabolism. This is, to our knowledge, the most comprehensive attempt at stoichiometric modeling of brain metabolism to date in terms of its coverage of a wide range of metabolic pathways. We then attempted to model the basal physiological behaviour and hypoxic behaviour of the brain cells where astrocytes and neurons are tightly coupled.

**Results:**

The reconstructed stoichiometric reaction model included 217 reactions (184 internal, 33 exchange) and 216 metabolites (183 internal, 33 external) distributed in and between astrocytes and neurons. Flux balance analysis (FBA) techniques were applied to the reconstructed model to elucidate the underlying cellular principles of neuron-astrocyte coupling. Simulation of resting conditions under the constraints of maximization of glutamate/glutamine/GABA cycle fluxes between the two cell types with subsequent minimization of Euclidean norm of fluxes resulted in a flux distribution in accordance with literature-based findings. As a further validation of our model, the effect of oxygen deprivation (hypoxia) on fluxes was simulated using an FBA-derivative approach, known as minimization of metabolic adjustment (MOMA). The results show the power of the constructed model to simulate disease behaviour on the flux level, and its potential to analyze cellular metabolic behaviour *in silico*.

**Conclusion:**

The predictive power of the constructed model for the key flux distributions, especially central carbon metabolism and glutamate-glutamine cycle fluxes, and its application to hypoxia is promising. The resultant acceptable predictions strengthen the power of such stoichiometric models in the analysis of mammalian cell metabolism.

## Background

Understanding of the biochemistry and energy metabolism of the brain is a prerequisite for evaluating the functioning of the central nervous system (CNS) as well as the physiology and pathology of the brain. The functions of the CNS are mainly excitation and conduction as reflected in the continuous electrical activity of the brain. The fact that this electrical energy is ultimately derived from chemical processes reveals the fundamental role of biochemistry in the operation of the brain.

Developments in functional brain imaging techniques have led to better elucidation of the physiological and biochemical mechanisms of the brain [1-4]. However, the exact mechanism still remains unknown. To simplify and interpret the actual metabolic mechanisms, mathematical models are commonly used as techniques to supplement the available experimental studies [5-9] where biochemical equations are solved in a systematic way to explain the missing physiological responses.

Brain energy metabolism has been approached by the use of dynamic modeling [5,8] where the main interaction takes place between the neuron and the blood stream. On the other hand, brain function depends on the coordinated activities of a multitude of cell types, such as neurons, astrocytes and microglia. Astrocytes play an important role in maintaining brain metabolism which, when disturbed, might lead to neurological diseases [10,11]. These two types of cells (i.e. neurons and astrocytes) are also important in neurotransmitter metabolism [12-14]. It was experimentally shown [1,10,11] that the interactions between neurons and their neighboring astrocytes required more thorough investigation [15-17] for a better understanding of the neurovascular and neurometabolic coupling specifically in pathological conditions. To date, it has proved a daunting task to identify all the metabolic pathways of brain energy metabolism and develop a dynamic simulation environment that will cover a time scale ranging from seconds to minutes to hours. To simplify this task and to make it more practicable, we undertook stoichiometric modeling of brain energy metabolism with the major aim of including all the known pathways between astrocytes and neurons.

We performed an extensive literature survey to obtain the catabolic, anabolic and exchange reactions in brain metabolism. Only about 100 references cited directly within the text are listed here. The ultimate goal was to develop a reliable stoichiometric model of the coupling mechanism, which will be compatible with physiological observations. The constructed model included central metabolism (glycolysis, pentose phosphate pathway, TCA cycle), amino acid metabolism (synthesis and catabolism), lipid metabolism, ROS detoxification pathway, neurotransmitter metabolism (dopamine, acetylcholine, norepinephrine, epinephrine, serotonine) as well as coupling reactions between astrocytes and neurons. The metabolic reactions were compartmentalized with respect to their localization in cells (astrocyte, neuron) to obtain a more realistic representation. Additionally, cofactor (NADH, NADPH, FADH_2_) localization in cytosol or mitochondria was reflected in the compiled reaction list. This is, to our knowledge, the first comprehensive attempt at stoichiometric modeling of brain metabolism in terms of its coverage of a wide range of metabolic pathways (214 reactions). Flux balance analysis (FBA), a steady-state metabolic modeling technique [18,19], was applied to the reconstructed model to seek answers to the following questions: i) how the available fuel is shared among different pathways of the brain, ii) which quantifiable astrocyte-neuron interactions can be identified under resting conditions, iii) whether the neurotransmitters are produced at maximal rate in these conditions, and iv) whether hypoxia, a very common causative factor associated with neurological diseases, can be explained by the stoichiometric modeling of neuron-astrocyte coupling. The constructed model was also used to identify the intermediary biochemical reactions and elements that participate in trafficking (eg. glutamate-glutamine, branched-chain amino acid shuttles) and to examine the interactions among the pathways. The predictions were verified by comparing corresponding flux distributions to literature findings from a pathway-oriented perspective.

## Results and Discussion

### Metabolic model reconstruction

**Figure 1:**
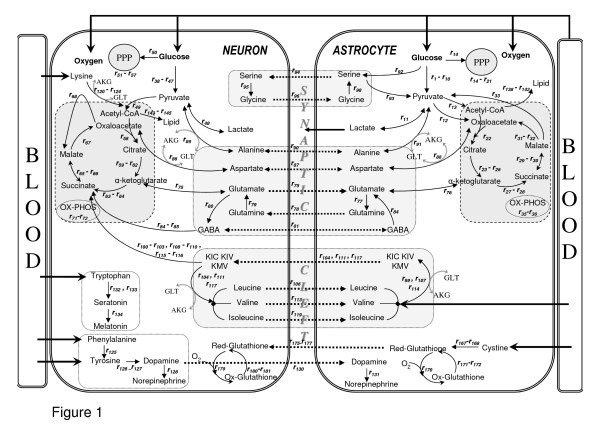
**Metabolic interactions between astrocytes and neurons with major reactions**. Thick arrows show uptake and release reactions. Dashed arrows indicate shuttle of metabolites between two cell types. Glutamate and α-ketoglutarate in transamination reactions are abbreviated as GLU and AKG, respectively. All reactions considered in the modeling are given in additional file 1. The reaction numbers in the figure refer to the numbering in the reaction list of additional file 1. Here we only depict major reactions for simplicity.

The main interaction site of neurons and astrocytes is known to be the synaptic cleft. Since both neurons and astrocytes require proximity to blood vessels for transmission of metabolites, a representation of this cellular organization is reconstructed (Figure 1). Although these interactions are known to occur in various time scales, the model assumes steady-state in metabolic pathways and guides us to investigate normal versus abnormal conditions of brain energy metabolism. Hence, we tried to incorporate as many of the pathways as possible into the model.

A previous attempt for stoichiometric modeling of brain metabolism [6] covered 16 reactions that mainly occur among glutamate and TCA cycle intermediates. That model was used to simulate the conditions where the glutamate-glutamine cycle was inactive. The present reconstruction, on the other hand, is an attempt to model the basal physiological behaviour of brain cells, where the cycle is known to be active, through tight coupling between astrocytes and neurons. Our reconstructed model therefore includes the well-known glutamate-glutamine cycle, as well as other metabolic couplings and neurotransmitter synthesis reactions for the first time in the literature. Hence, this is the most comprehensive stoichiometric brain model developed to date. The constructed stoichiometric model consists of 217 reactions (184 internal, 33 exchange) and 216 metabolites (183 internal, 33 external) distributed in and between astrocytes and neurons (Additional File 1). Seventy-eight of the internal reactions occur in astrocytes, and 90 of them are localized in neurons. A high percentage co-occur in both cell types. The fact that the remaining 16 reactions are intercompartmental indicates the coverage of neuron-astrocyte coupling mechanisms by the constructed model. Additional File 2: Supplementary Table 1 details the metabolic differences in the two cell types reflected in the model reactions. Thirty-one of the 216 metabolites are taken as extracellular since they are associated with either an uptake (glucose^A,N^, oxygen^A,N^, ammonia^A^, leucine^A^, isoleucine^A^, valine^A^, phenylalanine^N^, tryptophan^N^, lysine^N^, tyrosine^N^, linoleate^A,N^, linolenate^A,N^, choline^A,N^, cystine^A^) or a release (CO_2_^A,N^, lactate^A^, dopamine^N^, acetylcholine^N^, norepinephrine^N,A^, epinephrine^N^, melatonin^N^, serotonin^N^, glutamine^A^, glutathione^N^) mechanism. Additionally, synthesized lipids in both cell types were considered as released for the modeling purposes.

As proposed [20-23], the main energetic pathways of brain (glycolysis, PP pathway, TCA cycle and oxidative phosphorylation) were considered to occur in both cell types (r_1_–r_37_/r_38_–r_73_), except the pyruvate carboxylation reaction (r_12_), whose enzyme is known to be inactive in neurons [17,24]. That is why neurons cannot replenish their TCA cycle intermediates and their derivatives, including glutamate, from glucose on their own. Since cofactors cannot cross the mitochondrial membrane, their localization was reflected in the reactions. Accordingly, pyruvate dehydrogenation (r_13_, r_49_), is mitochondrial. Both NADH-(mitochondrial) and NADPH-dependent (mitochondrial and cytosolic) isocitrate dehydrogenation reactions (r_24_–r_26_/r_60_–r_62_) were taken into account [25]. Malic enzyme is confined to the cytosol in astrocytes (r_33_) whereas it is only mitochondrial in neurons (r_69_) [26-28]. The malate-aspartate shuttle plays an important role in neurons, by transferring reducing equivalents (NADH) from the cytosol to mitochondria for ATP synthesis through oxidative phosphorylation [29-33]. Accordingly, a cytosolic version of malate dehydrogenation (r_69_) in the reverse direction was included in neurons in addition to the mitochondrial version, to mimick the shuttle. In astrocytes, however, cytosolic malate dehydrogenation was considered in the same direction as the mitochondrial one since it is known that the malate-aspartate shuttle is not active in astrocytes [29,31], although cytosolic malate dehydrogenase is present in this cell type [34,35]. The mitochondrial transhydrogenase converting NADH to NADPH [36] was also considered. ATP consumption by the ATPase pumps and other processes (r_37_/r_73_) was also accounted for. Lactate release was assumed to be only from the astrocytes [37] since it is known that neuron metabolism is primarily oxidative.

An extensive literature survey was performed to acquire the compartmentation of amino acid catabolism and synthesis between astrocytes and neurons. For the glutamate – glutamine cycle (r_74_–r_79_) [38,39], glutamate is released from neurons and subsequently taken up by astrocytes and returned to neurons via synaptic clefts again in the form of glutamine. Unlike astrocytes, neurons cannot generate glutamine from glutamate owing to the lack of the glutamine synthetase enzyme [13]. They have glutaminase enzyme instead (r_79_) to convert astrocyte-derived glutamine into glutamate. One alternative for neuronal glutamate production is the transfer of TCA cycle intermediates from astrocytes to neurons. However, these exchange reactions were not added to the model since there is not sufficient evidence for such trafficking [13,16,40,41]. Since glutamate uptake by astrocytes activates Na^+^K^+^ATPase [42,43], the associated consumption of 1 ATP was included in the corresponding equation (r_75_). Glutamine efflux from the astrocytes to the extracellular space [7,44] was taken into account as well. Glutamate dehydrogenase is located in mitochondria, and this is reflected in the cofactor specification of the corresponding reactions (r_74_, r_76_) [45].

NMR studies indicate that the GABA, aspartate and alanine pathways are closely linked to the glutamate – glutamine cycle [40,46]. GABA is assumed to be formed by the decarboxylation of glutamate (r_80_) in neurons and then transferred into the neighboring glial cells where it is converted into glutamate and succinate irreversibly (r_81_–r_83_) [47,48]. Conversion to succinate is also possible in neurons (r_84_–r_85_) [49]. Aspartate can be formed both in astrocytes and neurons reversibly via transamination (r_86_, r_88_), and it can be transferred between the two cell types in both directions (r_87_) [40,47]. It has been claimed [50,51] that alanine is released by neurons, taken up by astrocytes and transformed into pyruvate and acts as a nitrogen carrier from neurons to astrocytes. On the other hand, it has been suggested [52] that alanine is produced and released by astrocytes for the use of neurons. To consider both possibilities, these reactions and transfer of alanine between the cell types were defined as reversible (r_89_–r_91_).

Serine and glycine are involved in a cycle between astrocytes and neurons analogous to the glutamate-glutamine cycle [53,54]. There is no 3-phosphoglycerate dehydrogenase activity in neurons; hence the corresponding reaction only occurs in astrocytes [55]. The cofactor localization of the reaction (r_92_) is cytosolic [56]. Once formed from glutamate and 3-phosphoglycerate in astrocytes (r_92_) [55-57], serine can be transported to neurons (r_94_), where it is converted to glycine (r_95_) [48,58]. Conversion of serine to pyruvate (r_93_) is also possible in astrocytes [53,58,59]. Neuronal glycine can be transported to astrocytes (r_96_) [48], where it is converted back to serine (r_98_), completing the cycle [54,58,60]. Additionally, the glycine cleavage system (r_97_) is exclusively active in astrocytes [54,61], and located in mitochondria.

Inclusion of branched chain amino acids (BCAA) in the model is crucial for the investigation of brain metabolism coupling and the glutamate – glutamine cycle because they serve as nitrogen donors for glutamate and transfer nitrogen from astrocytes to neurons [62-64]. BCAA metabolism is compartmented between astrocytes and neurons. Astrocytes take up leucine from the blood brain barrier [65] and oxidize it so as to form a branched chain keto acid, α-ketoisocaproate (KIC) (r_99_), to supply amino nitrogen to the glial glutamate pool. Then KIC is transferred into the neuronal compartment (r_104_) and converted back to leucine (r_105_). The cycle is finalized by the conveyance of leucine to the astrocyte (r_106_) [64,66]. It is also possible that leucine in the form of KIC enters the astrocytic TCA cycle as acetyl-CoA [64], as considered by the model (r_100_–r_103_). The other branched chain amino acids, valine and isoleucine, are associated with comparably lower uptake rates [67]. Their mechanisms in brain are essentially similar, except the last step where they are converted not to acetoacetyl-CoA but to succinyl-CoA (r_107_–r_119_) [64,68]. Branched chain keto acid dehydrogenase reactions (r_100_, r_108_, r_115_) take place in mitochondria [26,69,70], together with branched chain acyl-coa dehydrogenase reactions (r_101_, r_109_, r_116_) [26,68,71].

Lysine catabolism via the saccharopine pathway has been shown to occur mostly in neurons [72]. Hence, lysine was allowed to be taken up by neurons leading to glutamate production (r_120_–r_121_) and it was degraded to acetyl-CoA (r_122_–r_124_) [68,72]. The pathway is cytosolic until the formation of alpha-ketoadipate (r_121_), after which it takes place in mitochondria (r_123_) [68]. No astrocytic pathway was considered for lysine since there was no suggested mechanism for this cell type in the literature.

Phenylalanine taken up from the extracellular space is catabolized to tyrosine (r_125_) [48,73,74]. Tyrosine, coming from phenylalanine or transported from the blood, is converted to DOPA by tyrosine hydroxylase using oxygen in neurons, and this is eventually converted into the neurotransmitter dopamine (r_126_–r_127_) [48,75-77]. As the neurotransmitters are synthesized in neurons, uptake of the corresponding substrate, tyrosine, from the blood-brain barrier was assumed neuronal. This can be followed by norepinephrine and epinephrine syntheses (r_128_–r_129_). Dopamine can be released from neurons into the synaptic cleft or stored in vesicles [76]. Therefore, dopamine release to extracellular space was included in the model. Moreover, it has been reported that dopamine is taken up by astrocytes from the synaptic cleft and converted to norepinephrine [78]. This suggested metabolite trafficking was also taken into account in the model (r_130_–r_131_).

Tryptophan serves as a precursor for the synthesis of serotonin and melatonin in neurons following its uptake (r_132_–r_134_) [79]. Since serotonin is stored in vesicles, it is considered as extracellular. Acetylcholine as a neurotransmitter is synthesized from acetyl-CoA in neurons (r_135_) [48].

Although they are essential amino acids for brain, the catabolism of threonine and methionine was ignored because of their very low uptake rates [67].

The precursor for the synthesis of lipids is acetyl-CoA. The major lipid types are triacylglycerols, cholesterol, and phospholipids. Brain contains virtually no triacylglycerol [74,80]. Therefore, related synthesis pathways were not taken into account. All cholesterol in the brain is produced by local synthesis in astrocytes (r_136_) [81], with no supply from other organs [82]. Necessary cholesterol for neurons is supplied from astrocytes (r_137_), forming a cholesterol shuttle between the two cell types [81,83,84]. The lack of cholesterol synthesis in neurons in the adult state is probably due to its high energetic cost (r_136_).

The building blocks for phospholipids are fatty acids, which are synthesized from acetyl-CoA (r_140_–r_149_) in cytosol. Nonessential fatty acids (palmitate, oleate, stearate) are synthesized de novo in both cell types (r_140_–r_142_, r_145_–r_147_) [85]. Arachidonate and decosahexenoate, however, require uptake of the essential fatty acids linoleate and linolenate respectively by the astrocytes (r_141_–r_142_), which can be provided externally, eg. through diet. Neurons are not capable of producing these two fatty acids, instead they take up the ones synthesized and released by astrocytes (r_146_–r_147_) [86,87]. These five fatty acids constitute more than 90% of phospholipids [80,88], therefore other fatty acid types were ignored because of their very low percentage. Accordingly, fatty acid synthesis reactions in both cell types were written on the basis of the molar composition reported in [80] (r_148_–r_149_). The same composition was assumed for astrocytes and neurons since it has been reported that these two cell types have very similar fatty acid and lipid compositions [89]. Phospholipids are synthesized from fatty acids and glycerol-3-phosphate, which is a product of a dehydrogenation reaction (r_150_, r_158_) [74,75]. Here, phospholipids are assumed to be composed of phosphatidyl-choline, phosphatidyl-serine, and phosphatidyl-ethanolamine, which together constitute about 85% of brain phospholipids [74,80,89-91]. The related reactions (r_152_–r_157_, r_159_–r_164_) were compiled from [74,75,92]. Finally, the synthesis of lipid in both cell types was expressed in terms of reactions whose stoichiometric coefficients are based on the molar lipid compositions reported in [80] (r_165_–r_166_).

Glycerol-3-phosphate formation reaction is cytosolic in astrocytes (r_150_) [31], and mitochondrial in neurons (r_158_) [31,93]. Since the malate-aspartate shuttle is not active in astrocytes, another shuttle mechanism must be active in this cell type to transport cytosolic NADH produced due to a high rate of glycolysis to mitochondria. Following this logic, the glycerol-3-phosphate shuttle was proposed to be active in astrocytes [31], which is validated by the presence of cytosolic and mitochondrial versions of the enzyme in astrocytes [35]. Therefore, the dehydrogenation reaction in astrocytic mitochondria was added to the model in reverse direction (r_151_), allowing the transfer of cytosolic NADH to mitochondria in the form of FADH_2_.

The brain requires glutathione for the removal of reactive oxygen species (ROS) such as H_2_O_2_. Glutathione is synthesized from cysteine (r_168_, r_177_), which is derived from cystine (r_167_). Because only astrocytes can take cystine up from the blood vessel and convert it to cysteine, neurons are dependent on astrocytes for protection against oxidative stress [11,94,95]. In astrocytes, formed peroxides (r_169_) are removed by glutathione (r_170_). The resulting oxidized glutathione is converted back to the reduced form by glutathione reductase (r_171_, r_172_), which requires NADPH and is located in both cytosol and mitochondria [27]. Alternatively, catalase can convert peroxides back to oxygen in the brain (r_173_) [27]. Reduced glutathione can be converted to cysteinyl-glycine in astrocytes (r_174_), which is used as the cysteine supply to the neurons (r_175_, r_176_). Then cysteine acts as precursor for neuronal glutathione (r_177_). The following protection mechanism is the same as in astrocytes (r_178_–r_182_).

Brain has a high glycogen content [96], and astrocytes contain nearly all of it [97,98]. In normal physiological conditions, however, the rate of glycogen phosphorylation to glucose-6-phosphate and the rate of glycogen synthesis from glucose-6-phosphate were found to be equal [98]. That is, there is no net effect of glycogen on brain metabolism under normal physiological circumstances. Therefore, we do not include glycogen in modeling of the resting state. However, it is hypothesized that glycogen may act as a buffer under stress conditions such as hypoxia [98]. Therefore, astrocytic glycogen breakdown reactions were included in the model in such a way that they are only allowed to be active during hypoxia simulation (r_183_–r_184_).

Other pathways such as nucleotide metabolism were not taken into account since there is no detailed information on the compartmentation of those pathways between the two cell types, and no significant fluxes have been reported through such pathways. One should also note that an individual neuron may not have all the reactions detailed above since individual neurons are specialized to synthesize specific neurotransmitters. Here we consider a population of neurons rather than individuals, thereby aiming at the overall picture in the brain.

A hypothesis called ANLSH (astrocyte-neuron lactate shuttle hypothesis) proposes the use of astrocyte-derived lactate as energy substrate by neurons under activated conditions [99] where there is a stimulus. In the first part of our work, we model brain metabolism under resting conditions in the absence of any stimulus. That is why we did not consider any transfer of lactate from astrocytes to neurons in our model for the analysis of basal physiological behaviour. In the second part of the work, where we model hypoxic behaviour, the lactate shuttle is again not considered. The idea behind ANLSH is to supply lactate as an oxidative substrate for neurons to keep the TCA cycle active, as an energetic contribution to aerobic neuronal metabolism. However, the hypoxic state is associated with gradual inactivation of the TCA cycle with restricted aerobic metabolism. Additionally, neurons start to produce lactate in this state owing to reduced oxygen uptake. Therefore, neurons do not need to use astrocytic lactate since they already produce it. As a result, hypoxic analysis is performed without any lactate transfer between the two cell types.

### Model prediction: Flux distributions among key pathways

**Figure 2:**
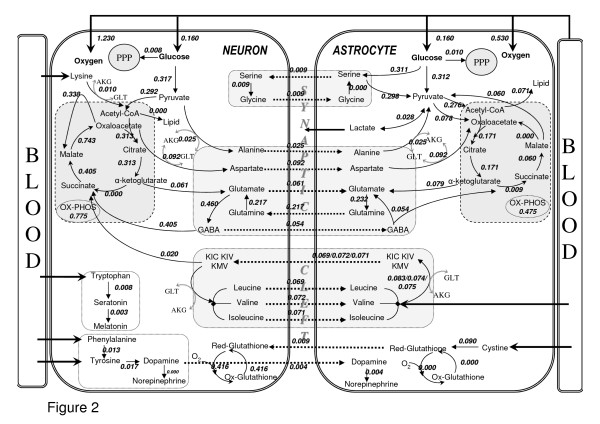
**Major metabolic fluxes (μmol/g tissue/min) in neuron-astrocyte coupling for resting conditions**. The fluxes were calculated with the objective of maximizing the glutamate/glutamine/GABA cycle fluxes between the two cell types with subsequent minimization of Euclidean norm of fluxes, using the uptake rates given in Table 1 as constraints. Thick arrows show uptake and release reactions. Dashed arrows indicate shuttling of metabolites between the two cell types. Only key pathway fluxes are represented here for simplicity. The flux distributions for all the reactions listed in Additional File 1 are given in Additional File 4:Supplementary Table 3.

The constructed model was first utilized to simulate the neuron-astrocyte flux distribution under resting conditions based on the constraints (Table 1) detailed in the Methods section. FBA using an objective function together with the imposed constraints is employed owing to the underdetermined nature of the reconstructed network, to get an optimum flux distribution (see Methods section). The common objective function of maximal biomass production used in FBA applications of unicellular cells can hardly be applied to multifunctional cells. Therefore, a number of objective functions as listed in Additional File 3: Supplementary Table 2 were employed and the one that gave best agreement with the literature data was identified. The major criteria used in the judgment of suitability of the objective functions were a) agreement with the literature-based lactate release flux, b) getting an active glutamate-glutamine cycle, c) getting active BCAA shuttles, and d) getting active fluxes for PPPs; as the related reactions have been extensively discussed in the literature. Simulations indicated that use of simultaneous maximization of glutamate/glutamine/GABA shuttling reactions between astrocytes and neurons (r_75_, r_78_, r_81_) with subsequent minimization of the Euclidean norm of fluxes result in a flux distribution in accordance with literature data. The following results and discussions are, therefore, based on this flux distribution. The deficits for other employed objective functions (the points where they contradict the used criteria) are given in Additional File 3: Supplementary Table 2. Using the successful objective function, flux results regarding the key pathways are depicted in Figure 2. Thus, the FBA results allowed us to identify how the available fuels (glucose, essential amino acids) are shared among the different pathways of the two cell types, as demonstrated in Figure 2 and discussed below.

**Table 1:** Blood-brain barrier uptake rates of glucose, oxygen, ammonia, cystine and essential amino acids; and carbon dioxide release rate (μmol/g tissue/min). The related references for the rates are given under "Parameters used in the stoichiometric model" section. A: Astrocytes, N: Neurons, CMR: Cerebral Metabolic Rate

CMR_Glucose_^A^	0.160
CMR_Glucoses_^N^	0.160
CMR_O2_^A^	0.530
CMR_O2_^N^	1.230
CMR_CO2_^A^	0.515–0.530
CMR_CO2_^N^	1.193–1.230
Cystine^A^	0.0045
Ammonia^A^	0.0035
Phenyalanine^N^	0.0132
Tryptophan^N^	0.0082
Leucine^A^	0.0145
Isoleucine^A^	0.0040
Tyrosine^N^	0.0041
Valine^A^	0.0018
Lysine^N^	0.0103

An additional table is provided (Table 2) which shows the maximum and minimum attainable values of the fluxes or flux ratios used for verification in the model. Thereby, it is shown that the model with the specified constraints is flexible enough to attain different flux values, and the chosen objective functions have enabled the calculated flux values/ratios to be in accordance with literature.

**Table 2:** Minimum and maximum attainable values for fluxes/flux ratios used in the model to verify the model compared to basal FBA and literature values. The results show that the model with the specified constraints is flexible enough to attain different flux values, but it was the chosen objective functions that resulted in flux values/ratios in accordance with literature. See the results & discussion part of the main text for detailed discussion of FBA results.

% Flux Ratio	minimum	maximum	FBA of resting state*	literature values in percentage
% Lactate release flux (r_11_) with respect to CMR_glc_	0	16	4.5/4.7	3–9 [105-108]
% Glutamate/Glutamine cycle flux (r_78_) with respect to CMR_glc_	0	68	68/56	40–80 [7; 44; 104]
r_TCA,A_/r_TCA,total_, r_22_/(r_22 _+ r_58_) (percent relative oxidative metabolism of astrocytes)	12	42	35/35.4	30^# ^[7; 97; 100]
% total lipid synthesis with respect to CMR_glc_	0.6	3.8	2.8/2.8	2 [74]
% total PPP flux with respect to CMR_glc_	0	5.6	5.6/5.6	3–6 [151; 152]
% pyruvate carboxylase flux (r_12_) with respect to CMR_glc_	2.8	45	11.7/10.8	10 [7; 100; 103]

* The second values in this column are results of resting state simulation with 40%–60% partitioning of glucose utilization between neurons and astrocyte respectively, corresponding to glucose uptake rates of 0.128 μmole/g/min and 0.192 μmole/g/min. The results show that the flux ratios are robust to the relative glucose uptake rates by the two cell types.

#The literature value for this percentage is based on experimental results on human [7] and rat [100] as reported in Table 1 and corresponding footnotes of [97]. However, others [156] calculated a lower percentage (19%) for human, based on the same experimental data.

#### Central Carbon metabolism

The ratio of neuronal TCA cycle flux to the total TCA cyle flux, r_22_/(r_22 _+ r_58_), is calculated as 0.35 by our approach, which is in good agreement with the literature-reported value of 30% [7,97,100]. This ratio also represents the relative oxidative metabolism of astrocytes. Therefore, our simulations support the view that, albeit lower than that of neurons, astrocytes have active oxidative metabolism under the nonstimulated conditions in parallel with the reported findings [97,101,102], rather than having only anaerobic metabolism or very low oxidative metabolism. On the other hand, the ratio of astrocytic ATP generation for ATPase pump and maintenance (r_37 _+ r_75_) to the total ATP generation rate is 0.27, indicating the degree of relative ATP production in both cells, as consistent with the above-stated fraction of oxidative metabolism. Additionally, the percentage of model-based pyruvate carboxylase flux (r_12_) with respect to CMR_glc _(11.7%) matches very well with reported results of around 10% [7,100,103]. This flux is only astrocytic and enables *de novo *synthesis of TCA cycle intermediates in this cell type. The flux through reaction, which represents the activity of the malate-aspartate shuttle in neurons by transferring NADH from cytosol to mitochondria (r_68_), is calculated as 0.34 μmole/g/min. The magnitude of this flux is reported to be similar to that of the flux through neuronal pyruvate dehydrogenase (r_49_) [7]. Our results support this relationship since the latter flux acquires a value of 0.29 μmole/g/min in our simulations. The high flux also emphasizes the view that the shuttle is of considerable importance to neurons [30,31], contributing to ATP synthesis by transferring NADH to mitochondria. It was reported that malic enzyme is only astrocytic in physiological conditions [44,63]. The calculated flux through the cytosolic malic enzyme of astrocytes is 0.06 whereas that through the mitochondrial one in neurons is zero, supporting the physiological findings. The ratio of the rates of total TCA cycle to total glucose consumption, (r_22 _+ r_58_)/CMR_glc_, is calculated as 1.51 by our approach, which is lower than the reported values of approximately 2 [7,104]. The reason behind this discrepancy is that the Acetyl-CoA requirement for biosynthetic routes, especially for lipid metabolism, was ignored in those studies although significant molar amount is needed for cholesterol (r_136_) and fatty acid (r_138_–r_140_, r_143_–r_145_) syntheses. That is, some portion of glycolytic Acetyl-CoA is diverted to lipid metabolism leading to lower TCA fluxes. Therefore, our simulation result is in accordance with the expectation that the ratio r_TCA,total_/CMR_glc _must be lower than 2.

The present model results suggest that NADPH production through the pentose phosphate pathway, r_14 _and r_50_, is at the specified boundaries for both cell types. Regarding the fluxes through the ROS pathway; the model calculates astrocytic peroxide formation rates as zero, implying that the pathway is inactive in this cell type. This is in accordance with the relatively lower oxidative metabolism in astrocytes. For neurons, however, there is significant peroxide formation, and hence glutathione is oxidized and then reduced to remove oxidative stress. NADPH used for oxidative stress reduction is 0.18 and 0.24 μmole/g/min in cytosol (r_180_) and in mitochondria (r_181_) respectively.

The lactate release rate was calculated as 8.9% of glucose flux. In terms of the carbon-mole, this stands for 4.5% of glucose carbon through the lactate route, which is in the vicinity of the reported values at rest [105-108]. This percentage becomes higher when higher leucine uptake rates are considered as reported by others [70,105].

#### Glutamate-Glutamine Cycle and Other Cycles

The neuronal and glial compartments are known to be the two major compartments of brain metabolism, and they are metabolically linked with the glutamate-glutamine cycle. This has led to detailed investigations of the flux through this cycle, because it represents the hallmark of cerebral metabolic compartmentation and it is closely linked to the Krebs cycle [22,104,109].

The ratio between the glutamate-glutamine cycle and the glucose consumption rate, r_78_/CMR_glc_, was calculated by FBA as 0.68, which is in the range of reported values (0.41–0.80) [7,44,104]. The ratio attains a value on the upper border of the literature results (0.81), when the GABA cycling flux is added to the glutamate-glutamine cycling flux. Thus, the constructed stoichiometric model leads to a reasonable prediction regarding the well-known glutamate-glutamine cycle, which is essential for the functioning and coupling of astrocytes and neurons and has been of deep interest for researchers in this area [7,24,104,110,111]. Additionally, it has been reported that glutamine efflux to the extracellular space from astrocytes ranges between 0.002 and 0.080 μmol/g/min [44]. The value calculated by the present model (0.011 μmol/g/min) is in agreement with this range.

The cycles other than the glutamate-glutamine cycle were calculated to have lower flux values. Serine-glycine cycling operates with a flux of 0.01 μmol/g/min. The flux through each of the BCAA cycles, which are directly linked to the glutamate pool, is about 33% of the glutamate-glutamine cycle flux. In this way, they contribute to the glutamate-glutamine cycle flux. This contribution for leucine alone was reported as 25–30% [112] in parallel with our predictions. For valine and isoleucine, however, the reported values are much lower [64]. Also, a much higher astrocytic transamination rate of leucine (r_99_) than the decarboxylation rate of KIC (r_100_) has been reported [64]. The ratio of these fluxes (r_99_/r_100_) obtained by the present model is more than 5, consistent with physiological expectation. The directions of the aspartate and alanine cycle were from neurons to astrocytes, contributing to the astrocytic glutamate pool, with fluxes of 0.092 and 0.025 μmol/g/min respectively. Unlike the alanine cycle, the aspartate cycle acquires a relatively higher flux, which needs to be confirmed by experimental studies.

The above-discussed FBA results show which metabolic interactions were active between astrocytes and neurons under resting states, and the redistribution of corresponding fluxes in both cell types is indicative of the relative activity of the interactions.

#### Lipid Metabolism

Inclusion of lipid metabolism is especially important for ATP, NADPH and Acetyl-CoA balances to be closed. The model-based fluxes indicate that lipid synthesis under steady state conditions is possible in astrocytes, with a rate corresponding to 2.8% of glucose flux. This is in accordance with the literature value [74], which reports that about 2% of the glucose flux goes to lipid metabolism. Our model does not calculate any flux through neuronal lipid metabolism. This implies either a deficit of the model or the absence of any significant lipid synthesis rate in mature neurons.

#### In silico Neurotransmitter Production Capabilities

**Figure 3:**
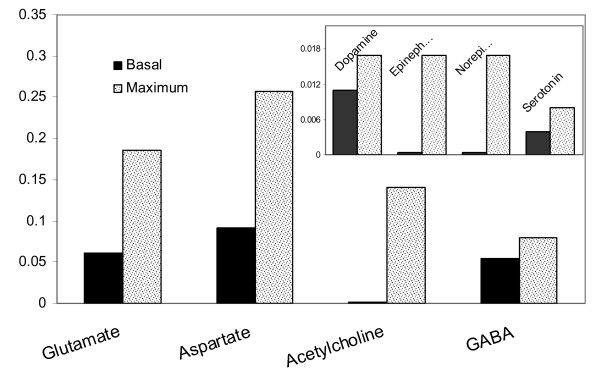
**Neurotransmitter production rates (μmole/g/min) under resting conditions in comparison with their maximum values**. The rates for resting conditions were calculated with the objective maximizing glutamate/glutamine/GABA cycle fluxes between the two cell types with subsequent minimization of Euclidean norm of fluxes. The maximum value that a neurotransmitter production flux can attain was calculated for comparison by maximizing each of these fluxes one by one using linear programming.

To identify the maximum production capabilities of the brain cells for the major neurotransmitters, FBA was applied to the constructed stoichiometric model using the maximization of each of these neurotransmitters as the objective function. The resultant fluxes were compared with those obtained in the simulation of the resting condition analyzed above. Since neurotransmitters are produced in neurons and released to synaptic clefts, the flux values of the reactions that carry them from neurons to the extracellular space, or to astrocytes to clear them from synaptic clefts, were used in the analysis. Figure 3 depicts the results comparatively. Aspartate has the highest production rate under resting conditions followed by glutamate and GABA, whereas all the others have minute fluxes. Serotonin, GABA and dopamine were found to be synthesized at rates close to their theoretical maxima. For all the remaining neurotransmitters, the maximum production capability was several folds higher than their basal levels. For glycine, no finite maximum value could be identified, which implies partial uncoupling of this pathway from the rest of the network. Additional experimental and/or clinical research is necessary to verify these *in silico *predictions.

### Potential of the reconstructed model in the analysis of neural diseases

Many diseases of the brain have been reported to result from neurovascular coupling disorders, where mainly oxygen deficiency leads to a cascade of events. A decrease in cerebral perfusion due to arterial obstruction (loss of arterial compliance) leads to the formation of hypoxic regions in the brain as encountered in the pathophysiology of aging and several psychiatric disorders as well as headache. Hypoxic regions in the brain have been known to cause major disturbances in the electrical activity of the brain (as in epilepsy) or lead to progressive diseases such as dementia, Alzheimer's and even emotional disturbances. Hence, as a good predictor of our model, we chose to simulate the effects of hypoxia in hope that it can be explained by stoichometric modeling approaches.

**Figure 4:**
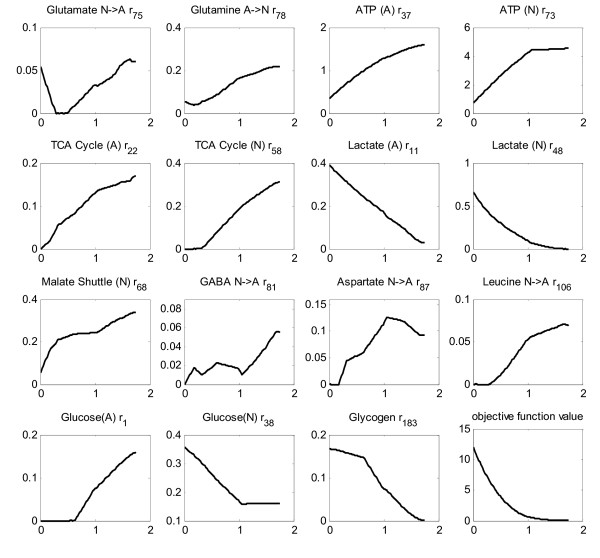
**Cerebral hypoxia**. Effect of oxygen deprivation of brain cells on metabolic fluxes calculated by MOMA approach. All the x-axes represent the oxygen flux, CMR_O2_, available to brain cells. It is changed from anoxic level (no oxygen uptake) to the basal level (1.760 μmole/g/min). The title of each sub-figure includes the reaction number of the plotted flux, as given in Additional File 1.

**Figure 5:**
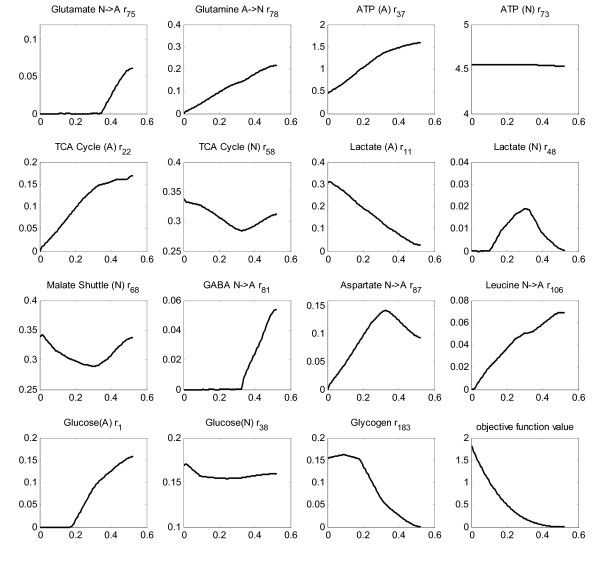
**Astrocytic hypoxia**. Effect of oxygen deprivation of astrocytes on metabolic fluxes calculated by MOMA approach. All the x-axes represent the oxygen flux available to astrocytic cells. It is changed from anoxic level (no oxygen uptake) to the basal level (0.53 μmole/g/min). (no lactate release from neurons). The title of each sub-figure includes the reaction number of the plotted flux, as given in Additional File 1.

It has been reported that deficient cells exhibit a flux profile closest to the healthy (non-deficient) flux distribution [113,114]. This finding was used as a basis to simulate oxygen deprivation of cerebral and astrocytic metabolism. Oxygen flux was gradually decreased in small intervals, and the new flux distributions were calculated using quadratic programming with the objective function of minimizing the Euclidean distance from the flux distribution of the healthy case, an approach called Minimization of Metabolic Adjustment, MOMA [114]. Glycogen breakdown reactions were made active in hypoxic simulations [98]. None of the fluxes in Table 1 that were used as constraints in the analysis of resting conditions were used in the simulation of hypoxia. Thereby, the effects of hypoxia on the uptake rates were also accounted for. Additionally, the flux through the pentose phosphate pathway in both cell types and GABA flux as well as RQ were left unconstrained. The only constraint was due to MOMA, i.e. obtaining a flux distribution as close to the healthy-case flux distribution as possible. The changes of the major fluxes in response to oxygen uptake deficiency are depicted in Figures 4 and 5. Such a simulation reflects the effect of hypoxic conditions on brain metabolism. A lactate efflux by neurons was considered in these simulations since oxygen deprivation results in the activation of anaerobic metabolism in this cell type.

Simulation of cerebral hypoxia (up to zero CMRO2) reveals more than tripling of astrocytic lactate production as well as significant neuronal production, implying the sharp activation of anaerobic metabolism (Figure 4). That is why the TCA cycle in both cells is found to exhibit a parallel gradual inactivation. In fact, these are the general characteristics of hypoxic cells) [115,116]. The decrease in malate shuttling rate in neurons (r_68_) in response to lower oxygen uptake flux, as shown in Figure 4, means less cytosolic NADH contribution to oxidative phosphorylation in mitochondria, another indication of hypoxic functioning. This is in accordance with the finding that inhibition of malate shuttling substantially reduces oxidative metabolism in neurons [117]. On the other hand, there is almost a doubling of glucose uptake rate through neurons, whereas the astrocytic uptake rate becomes zero at about 65% oxygen deficiency, a result that needs verification. Increased glucose uptake rates during hypoxia to maintain ATP levels have been reported [118,119]. Increase in the glucose uptake rate during anoxia was also reported as a mechanism to cope with Alzheimer's disease [120]. The flux through astrocytic glycogen breakdown becomes active in the initial phase of the hypoxic state, and increases considerably as the anoxic state is approached. The importance of glycogen metabolism in hypoxia has been stated [96]. One other clear feature is the impairment of the transfer flux of glutamate from neurons to astrocytes (r_75_) and the concurrent significant decrease in the return part of the cycle flux (r_78_). Such an impairment has already been described [10]. The significantly decreased flux through the glutamate-glutamine and alanine cycles and the ceased fluxes of the BCAA, Aspartate and GABA cycles are consistent with the hypothesis that hypoxia leads to lower trafficking between astrocytes and neurons)[115]. The abrupt effect of oxygen deficiency on brain metabolism is also reflected by the huge change in the objective function value (Figure 4).

Since the effect of hypoxia on only astrocytes was studied in detail [116], their oxygen deprivation was simulated separately here, by providing neurons with the baseline oxygen flux and restricting astrocytic oxygen uptake. The effect of hypoxia on astrocytic cells (Figure 5) is found to be relatively mild, as manifested by the magnitude of change of the objective function value. That is, astrocytes cope more readily with hypoxia than neurons, as demonstrated by several researchers [116,121,122]. Simulations indicate that these cells have the potential to function anaerobically (with no oxygen flux), which is already known as one of their characteristics [123,124]. However, glutamate transfer from neurons to astrocytes stops functioning after a certain level of allowed CMRO2 (0.35 μmole/g/min), similar to that observed in the simulation of cerebral hypoxia. This, compared with the results above, suggests that it is the degree of oxidative metabolism of the astrocytes that monitors the activity of the cycle. In other words, although no perturbation was applied to neurons, astrocytic oxygen deprivation led to the cessation of the uptake of neuronal glutamate by astrocytes. Simulation of neuronal hypoxia (results not shown) confirms this *in silico *prediction since a significant increase, rather than a decrease, in glutamate uptake rates by astrocytes was calculated in this case. In addition, results suggest relative uncoupling of ATP production mechanisms of the two cells since the neuronal ATP production rate and TCA cycle rate are found to be almost unaffected (Figure 5). Interestingly, astrocytic hypoxia triggers neuronal anaerobic metabolism as manifested by the low lactate flux (max. 0.02 μmole/g/min). One simulation result that conflicts with literature results is a decrease rather than an increase in astrocytic glucose uptake flux in response to hypoxic stress. Instead, an increase in glycogen breakdown flux is observed in astrocytes. Nevertheless, the literature data are for independent cultivation of astrocytes without neurons [116,121,122]. Here, we simulate astrocytic hypoxia in the presence of neurons, which can work as a metabolic support for astrocytes preventing an increase in their glucose uptake rate.

The present results demonstrate the power of the constructed model to simulate disease behaviour on the flux level, and its potential to analyze cellular metabolic behaviour *in silico*. Preliminary analysis of some other common metabolic diseases such as hyperammonaemia, maple syrup urine disease and phenylketonuria by this approach is also promising (unpublished results).

## Conclusion

Stoichiometric flux analysis techniques have been successfully applied to the analysis of mammalian cells [6,125-128]. Compared to a previous attempt at stoichiometric modeling of brain metabolism [6], the reconstructed model presented here not only includes the well-known glutamate-glutamine cycle, but also takes BCAA coupling, aspartate, alanine, serine, glycine, glutathione and GABA couplings, and neurotransmitter and lipid synthesis reactions, into consideration as well, for the first time in the literature. We thus attempted to model the basal physiological behaviour of brain cells where astrocytes and neurons are tightly coupled. By employing a reasonable objective function (simultaneous maximization of the GABA/Glutamate/Glutamine cycle fluxes with subsequent minimization of the sum of the fluxes) we have obtained flux values/ratios in accordance with the literature. The predictive power of the constructed model for the key flux distributions, especially central carbon metabolism and the glutamate-glutamine cycle fluxes, and for the capabilities of neuron and astrocyte metabolism is promising. This model can additionally be used in the analysis of metabolic neurological diseases since the use of similar stoichiometric modeling approaches for metabolic diseases has already been demonstrated [113,125,129,130]. Such models also have the potential to be used for hypothesis testing. Although the present stoichiometric model gave some unexpected results, such as the prediction of a high aspartate flux between the two cell types, such limitations are already known for stoichiometric models that only depend on the stoichiometry of reactions and measured uptake fluxes as constraints, and therefore the regulatory events occurring in the cell cannot be incorporated. Integration of regulation into such models has been attempted [131,132], but the appropriate method for such implementation still remains to be established.

## Methods

### Computational protocol

The stoichiometric coefficients of the compiled reactions and their reversibility information were used to constrain the flux solution space;

*S *× *v *= 0

*v*_min _≤ *v *≤ *v*_max_

where *S *is an *m *by *n *stoichiometric matrix with *m *being the number of metabolites (213) and *n *being the number of reactions covering the pathway and the exchange reactions (214), *v *is the flux vector to be identified, and *v*_*min *_&*v*_*max *_are the lower and upper bounds of the fluxes based on the reversibility information. The uptake rates of extracellular metabolites (Table 1) from the blood brain barrier were all specified to constrain the model further. To this aim, the lower and upper bounds of the corresponding reactions were set to the reported values given in Table 1. The degrees of freedom of the reconstructed reaction network, *S*, show the difference between the number of independent equations (i.e. independent metabolites; also equals to the rank of matrix S) and number of unknowns (i.e. reaction rates) in the linear equation system defined by equation (1), and it is calculated as 47. Literature values of 15 fluxes (Table 1) were specified as constraints in FBA, resulting a remaining degrees of freedom of 32. Flux balance analysis (FBA), a solution technique for underdetermined systems that utilizes linear or quadratic programming to find an optimum solution [133], was used to identify metabolic flux distributions in and between the two types of brain cells based on the applied constraints. To express this mathematically,

min f ^T^*v*

subject to equations (1) and (2), with all entries of the row vector, f, being zero except the entries corresponding to the fluxes to be maximized. FBA has so far been mainly applied to microbial cells, for which biomass growth was used as standard objective function in the optimization problem. For mammalian metabolism, however, there is no standard objective function. Therefore, the optimum solution for this problem was sought under different candidate objective functions that are listed in Additional File 3: Supplementary Table 2 with corresponding reasonings for their selection. Thereby, we tried to identify a suitable objective function for brain metabolism.

On the other hand, in the FBA approach, there is the possibility of multiple optima, different flux distributions with the same optimal objective flux [134,135]. To eliminate the multiplicity of flux values due to this problem, the system was subjected to a second optimization: the minimization of the squared sum of all fluxes, known as minimization of the Euclidean norm, was used as an second objective function for FBA to ensure efficient channeling of all the fluxes through all pathways [136]. That is, by fixing the objective function value of the first optimization; minimization of sum of fluxes was applied, thereby selecting the flux distribution with the smallest sum of fluxes among all alternate optima. To express the second optimization mathematically,

$$\min\sum_{i}v_{i}^{2}$$

subject to the constraints by equations (1), (2), and additionally to the constraint through the first optimization:

f ^T^*v *= *objfun*

where *objfun *is the optimal value of the objective function obtained in the linear optimization problem of (3).

The underlying hypothesis is that cells aim to fulfill their functions with minimal effort since increasing the flux through any reaction will require an extra investment such as increasing enzyme levels [137]. Derivatives of this approach have been proposed and shown to be a suitable objective function for mammalian [137] and bacterial [138] cells.

Optimizations were performed in the MATLAB 7.0 environment with a MATLAB interface to CLP solver [139]developed by Johan Löfberg [140]. Flux values of all reactions in the resting state with the identified objective function, and in the anoxic state (complete lack of oxygen uptake), are given in Additional File 4: Supplementary Table 3. Matlab routines used in the simulations are available as Additional File 5.

### Parameters used in the stoichiometric model

Literature-based uptake fluxes of glucose, oxygen, ammonia, cystine and essential amino acids as well as carbon dioxide release flux (Table 1) were used as constraints on the reconstructed model in the simulation of fluxes by FBA.

Glucose and oxygen are the main substrates fueling both astrocytes and neurons, and their cerebral metabolic rates (CMR) are closely related. The arithmetic average of literature-reported cerebral glucose utilization rates for human brain under resting conditions (0.32 μmol/g tissue/min) [7,74,104,141,142] was used in the model. Many studies have reported the ratio of CMR_O2_/CMR_glc _in normal resting brain as about 5.5 [37,74,143]. Hence, the cerebral oxygen uptake rate was taken as 1.76 μmol/g tissue/min. Regarding individual uptake rates of glucose by neurons and astrocytes, it has been reported that about half of blood-borne glucose phosphorylation takes place in astrocytes in vivo [97,144]. Therefore, equal glucose uptake rates are assumed for each cell type. Thirty percent of total oxygen consumption in brain cortex is attributed to astrocytic cells [7,44,97]. Individual oxygen uptake rates of neurons and astrocytes were calculated on the basis of this percentage. Additionally, since it is known that the respiratory quotient (rCO_2_/rO_2_) of brain is very close to one with values reported between 0.91–1.00 [108,145], the CO_2 _production rate is constrained so as to give the reported RQ range for both cell types.

Amino acid uptake rates [67] were compartmentalized as discussed in the main text and listed in Table 1. For ammonia, the astrocyte is assumed to be the compartment for the uptake mechanism [146-148], and a rate of 0.0035 μmol/g tissue/min has been reported [149]. The cystine uptake rate was calculated from the arteriovenous concentration difference (9 μmol/L, [150]) and the cerebral blood flow rate in humans (0.50 ml/g/min, [142]). Two more constraints were added to the model to obtain more reliable flux distributions by reducing the solution space. The flux through the pentose phosphate pathway for astrocytes has been reported as 6% of the glucose consumption flux [151], while values up to 5% have been reported for neurons [152,153]. These percentages were used as the upper bounds for the flux through this pathway. Additionally, the reports [154,155] that GABA cycling flux is about 25% of glutamate-glutamine cycle flux were employed to constrain r_82 _as 25% of r_78_. This enabled the GABA pathway to be more tightly coupled with the overall network.

All the reported rates compiled from the literature (Table 1) are used as the input fluxes to the constructed model, and they are used as the constraints on the method employed, FBA, to obtain the corresponding metabolic flux distributions subject to the selected objective function. Since the degrees of freedom of the model is very high (47), we can safely specify/constrain a number of fluxes without harming the underdetermined nature of the metabolic system. Indeed, Table 2 gives the maximum and minimum attainable values of the fluxes or flux ratios used for verification in the model, indicating that the model with the specified constraints is flexible enough to attain different flux values. Specifying as many unknowns as possible is also necessary owing to the availability of alternate optima in such models, which otherwise may result in ambiguous values for some fluxes in the model.

The rates were compiled from different sources since it was not possible to obtain all measurements from a single source. Studies reporting analysis of these rates in the same system, which will be facilitated by novel high throughput measurement techniques, will lead to more accurate results, strengthening the role of such models in medical modeling.

## Abbreviations

FBA: Flux balance analysis, 

MOMA: Minimization of metabolic adjustment, 

CNS: Central nervous system, 

GABA: γ-aminobutyric acid, 

TCA: Tricarboxylic acid, 

CoA: Coenzyme-A, 

DOPA: Dihydroxyphenylalanine, 

CMR: Cerebral metabolic rate, 

BCAA: Branched chain amino acids, 

KIC: α-ketoisocapriate, 

KIV: α-ketoisovalerate, 

KMV: α-keto-β-methylvalerate, 

ROS: Reactive oxygen species, 

PPP: Pentose phosphate pathway, 

RQ: Respiratory Quotient, 

OX-PHOS: Oxidative Phosphorylation.

## Competing interests

The author(s) declare that they have no competing interests.

## Authors' contributions

KOU and AA designed the research. TC and SA reconstructed the model and performed the simulations. TC, SA, HS, AA and KOU analyzed the results. TC, SA, AA and KOU wrote the manuscript. All authors read and approved the final manuscript.

## Supplementary Material

Additional file 1 Reaction Set of Metabolic Reconstruction for Astrocytes and Neurons. Click here for file

Additional file 2 Supplementary Table 1 – Metabolic differences in two cell types reflected in our model. Click here for file

Additional file 3 Employed objective functions with corresponding reasonings. Click here for file

Additional file 4 Supplementary Table 3 – Flux distributions for all reactions in resting and anoxic states. Click here for file

Additional file 5 Matlab routines used in the simulations. Click here for file
