# Supplementary material for: Reconstruction and flux analysis of coupling between metabolic pathways of astrocytes and neurons: application to cerebral hypoxia
Source: Theor Biol Med Model. 2007 Dec 10;4:48. doi: 10.1186/1742-4682-4-48 (PMC2246127; doi:10.1186/1742-4682-4-48)
Supplement: Additional file 1 — Reaction Set of Metabolic Reconstruction for Astrocytes and Neurons. [file 1742-4682-4-48-S1.pdf]

Additional File 1: Reaction Set of Metabolic Reconstruction for Astrocytes and Neurons.

In metabolite names as suffix, A: astrocytic, N: neuronal. For cofactors: M indicates mitochondrial localization of the corresponding reaction. If there is no M, this means that the reaction is located in cytosol. 'EX' as suffix for metabolite names indicates extracellular version of that metabolite.

GLYCOLYSIS (ASTROCYTES)

R1a : Glucose_A + ATP_A → Glucose-6-phosphate_A + ADP_A

R2a : Glucose-6-phosphate_A ↔ Fructose-6-phosphate_A

R3a : Fructose-6-phosphate_A + ATP_A → Fructose-1-6- biphosphate_A + ADP_A

R4a : Fructose-1-6- biphosphate_A ↔ Glyceraldehyde-3-phosphate_A + Dihydroxyacetone-phosphate_A

R5a : Dihydroxyacetone-phosphate_A ↔ Glyceraldehyde-3-phosphate_A

R6a : Glyceraldehyde-3-phosphate_A + NAD_A ↔ 1-3-biphosphoglycerate_A + NADH_A

R7a : 1-3-biphosphoglycerate_A + ADP_A ↔ 3-phosphoglycerate_A + ATP_A

R8a : 3-phosphoglycerate_A ↔ 2-phosphoglycerate_A

R9a : 2-phosphoglycerate_A ↔ Phosphoenol-pyruvate_A

R10a : Phosphoenol-pyruvate_A + ADP_A → Pyruvate_A + ATP_A

R11a : Pyruvate_A + NADH_A ↔ Lactate_A + NAD_A

R12a : Pyruvate_A + ATP_A + CO2_A → Oxoloacetate_A + ADP_A

R13a : Pyruvate_A + NAD_AM → Acetyl-CoA_A + CO2_A + NADH_AM

PENTOSE PHOSPHATE PATHWAY (ASTROCYTES)

R14a : Glucose-6-phosphate_A + NADP_A → 6-phosphoglucone_A + NADPH_A

R15a : 6-phosphoglucone_A ↔ 6-phosphogluconate_A

R16a : 6-phosphogluconate_A + NADP_A → Ribulose-5-phosphate_A + NADPH_A + CO2_A

R17a : Ribulose-5-phosphate_A ↔ Xylulose-5-phosphate_A

R18a : Ribulose-5-phosphate_A ↔ Ribose-5-phosphate_A

R19a : Ribose-5-phosphate_A + Xylulose-5-phosphate_A ↔ Sedoheptulose-7-phosphate_A + Glyceraldehyde-3-phosphate_A

R20a : Sedoheptulose-7-phosphate_A + Glyceraldehyde-3-phosphate_A ↔ Fructose-6-phosphate_A + Erythrose-4-phosphate_A

R21a : Xylulose-5-phosphate_A + Erythrose-4-phosphate_A ↔ Fructose-6-phosphate_A + Glyceraldehyde-3-phosphate_A

#TCA CYCLE (ACTROCYTES)

R22a : Oxoloacetate_A + Acetyl-CoA_A → Citrate_A

R23a : Citrate_A ↔ Isocitrate_A

R24a : Isocitrate_A + NAD_AM → alpha-ketoglutarate_A + NADH_AM + CO2_A

R25a : Isocitrate_A + NADP_A → alpha-ketoglutarate_A + NADPH_A + CO2_A

R26a : Isocitrate_A + NADP_AM → alpha-ketoglutarate_A + NADPH_AM + CO2_A

R27a : alpha-ketoglutarate_A + NAD_AM → Succinyl-CoA_A + NADH_AM + CO2_A

R28a : Succinyl-CoA_A + ADP_A ↔ Succinate_A + ATP_A

R29a : Succinate_A + FAD_AM ↔ Fumarate_A + FADH2_AM

R30a : Fumarate_A ↔ Malate_A

R31a : Malate_A + NAD_AM → Oxoloacetate_A + NADH_AM

R32a : Malate_A + NAD_A → Oxoloacetate_A + NADH_A

R33a : Malate_A + NADP_A → Pyruvate_A + NADPH_A + CO2_A

R34a : NADP_AM + NADH_AM → NADPH_AM + NAD_AM

#OXIDATIVE PHOSPHORYLATION AND ATPASE (ASTROCYTES)

R35a : 6 ADP_A + 2 NADH_AM + 1 O2_A → 6 ATP_A + 2 NAD_AM

R36a : 4 ADP_A + 2 FADH2_AM + 1 O2_A → 4 ATP_A + 2 FAD_AM

R37a : ATP_A → ADP_A

GLYCOLYSIS (NEURONS)

R38n : Glucose_N + ATP_N → Glucose-6-phosphate_N + ADP_N

R39n : Glucose-6-phosphate_N ↔ Fructose-6-phosphate_N

R40n : Fructose-6-phosphate_N + ATP_N → Fructose-1-6- biphosphate_N + ADP_N

R41n : Fructose-1-6- biphosphate_N ↔ Glyceraldehyde-3-phosphate_N + Dihydroxyacetone-phosphate_N

R42n : Dihydroxyacetone-phosphate_N ↔ Glyceraldehyde-3-phosphate_N

R43n : Glyceraldehyde-3-phosphate_N + NAD_N ↔ 1-3-biphosphoglycerate_N + NADH_N

R44n : 1-3-biphosphoglycerate_N + ADP_N ↔ 3-phosphoglycerate_N + ATP_N

R45n : 3-phosphoglycerate_N ↔ 2-phosphoglycerate_N

R46n : 2-phosphoglycerate_N ↔ Phosphoenol-pyruvate_N

R47n : Phosphoenol-pyruvate_N + ADP_N → Pyruvate_N + ATP_N

R48n : Pyruvate_N + NADH_N ↔ Lactate_N + NAD_N

R49n : Pyruvate_N + NAD_NM → Acetyl-CoA_N + CO2_N + NADH_NM

PENTOSE PHOSPHATE PATHWAY (NEURONS)

R50n : Glucose-6-phosphate_N + NADP_N → 6-phosphoglucone_N + NADPH_N

R51n : 6-phosphoglucone_N ↔ 6-phosphogluconate_N

R52n : 6-phosphogluconate_N + NADP_N → Ribulose-5-phosphate_N + NADPH_N + CO2_N

R53n : Ribulose-5-phosphate_N ↔ Xylulose-5-phosphate_N

R54n : Ribulose-5-phosphate_N ↔ Ribose-5-phosphate_N

R55n : Ribose-5-phosphate_N + Xylulose-5-phosphate_N ↔ Sedoheptulose-7-phosphate_N + Glyceraldehyde-3-phosphate_N

R56n : Sedoheptulose-7-phosphate_N + Glyceraldehyde-3-phosphate_N ↔ Fructose-6-phosphate_N + Erythrose-4-phosphate_N

R57n : Xylulose-5-phosphate_N + Erythrose-4-phosphate_N ↔ Fructose-6-phosphate_N + Glyceraldehyde-3-phosphate_N

TCA CYCLE (NEURONS)

R58n : Oxoloacetate_N + Acetyl-CoA_N → Citrate_N

R59n : Citrate_N ↔ Isocitrate_N

R60n : Isocitrate_N + NAD_NM → alpha-ketoglutarate_N + NADH_NM + CO2_N

R61n : Isocitrate_N + NADP_NM → alpha-ketoglutarate_N + NADPH_NM + CO2_N

R62n : Isocitrate_N + NADP_N → alpha-ketoglutarate_N + NADPH_N + CO2_N

R63n : alpha-ketoglutarate_N + NAD_NM → Succinyl-CoA_N + NADH_NM + CO2_N

R64n : Succinyl-CoA_N + ADP_N ↔ Succinate_N + ATP_N

R65n : Succinate_N + FAD_NM ↔ Fumarate_N + FADH2_NM

R66n : Fumarate_N ↔ Malate_N

R67n : Malate_N + NAD_NM → Oxoloacetate_N + NADH_NM

R68n : Oxoloacetate_N + NADH_N → Malate_N + NAD_N

R69n : Malate_N + NADP_NM → Pyruvate_N + NADPH_NM + CO2_N

R70n : NADP_NM + NADH_NM → NADPH_NM + NAD_NM

OXIDATIVE PHOSPHORYLATION AND ATPASE (NEURONS)

R71n : 6 ADP_N + 2 NADH_NM + 1 O2_N → 6 ATP_N + 2 NAD_NM

R72n : 4 ADP_N + 2 FADH2_NM + 1 O2_N → 4 ATP_N + 2 FAD_NM

R73n : ATP_N → ADP_N

GLUTAMATE – GLUTAMINE CYCLE

R74n : alpha-ketoglutarate_N + NH3_N + NADPH_NM ↔ Glutamate_N + NADP_NM

R75c : Glutamate_N + ATP_A → Glutamate_A + ADP_A

R76a : alpha-ketoglutarate_A + NH3_A + NADPH_AM ↔ Glutamate_A + NADP_AM

R77a : Glutamate_A + NH3_A + ATP_A → Glutamine_A + ADP_A

R78c : Glutamine_A → Glutamine_N

R79n : Glutamine_N → Glutamate_N + NH3_N

GABA CYCLE

R80n : Glutamate_N → GABA_N + CO2_N

R81c : GABA_N ↔ GABA_A

R82a : GABA_A + alpha-ketoglutarate_A → Glutamate_A + SuccinateSAL_A

R83a : SuccinateSAL_A + NAD_AM → NADH_AM + Succinate_A

R84n : GABA_N + alpha-ketoglutarate_N → Glutamate_N + SuccinateSAL_N

R85n : SuccinateSAL_N + NAD_NM → NADH_NM + Succinate_N

ASPARTATE METABOLISM

R86n : Oxoloacetate_N + Glutamate_N ↔ Aspartate_N + alpha-ketoglutarate_N

R87c : Aspartate_N ↔ Aspartate_A

R88a : Aspartate_A + alpha-ketoglutarate_A ↔ Oxoloacetate_A + Glutamate_A

ALANINE METABOLISM

R89n : Pyruvate_N + Glutamate_N ↔ alpha-ketoglutarate_N + Alanine_N

R90c : Alanine_N ↔ Alanine_A

R91a : Alanine_A + alpha-ketoglutarate_A ↔ Pyruvate_A + Glutamate_A

GLYCINE-SERINE METABOLISM (tetrahydrofolate and methylenetetrahydrofolate are not included in the stoichiometric matrix)

R92a : 3-phosphoglycerate_A + Glutamate_A + NAD_A → Serine_A + alpha-ketoglutarate_A + NADH_A
R93a : Serine_A → Pyruvate_A + NH3_A
R94c : Serine_A → Serine_N
R95n : Serine_N → Glycine_N
R96c : Glycine_N → Glycine_A
R97a : Glycine_A + NAD_AM → CO2_A + NH3_A + NADH_AM
R98a : Glycine_A → Serine_A

LEUCINE METABOLISM

R99a : Leucine_A + alpha-ketoglutarate_A → KIC_A + Glutamate_A
R100a : KIC_A + NAD_AM → Isovaleryl-CoA_A + CO2_A + NADH_AM
R101a : Isovaleryl-CoA_A + FAD_AM + CO2_A + ATP_A → Acetoacetate_A + Acetyl-CoA_A + FADH2_AM + ADP_A
R102a : Acetoacetate_A + Succinyl-CoA_A → Acetoacetyl-CoA_A + Succinate_A
R103a : Acetoacetyl-CoA_A → 2 Acetyl-CoA_A
R104c : KIC_A → KIC_N
R105n : KIC_N + Glutamate_N → Leucine_N + alpha-ketoglutarate_N
R106c : Leucine_N → Leucine_A

VALINE METABOLISM (r110a is shared with isoleucine metabolism)

R107a : Valine_A + alpha-ketoglutarate_A → KIV_A + Glutamate_A
R108a : KIV_A + NAD_AM → Isobutyryl-CoA_A + CO2_A + NADH_AM
R109a : Isobutyryl-CoA_A + FAD_AM + 2 NAD_AM → FADH2_AM + 2 NADH_AM + CO2_A + Propionyl-CoA_A
R110a : Propionyl-CoA_A + CO2_A + ATP_A → Succinyl-CoA_A + ADP_A
R111c : KIV_A → KIV_N
R112n : KIV_N + Glutamate_N → Valine_N + alpha-ketoglutarate_N
R113c : Valine_N → Valine_A

ISOLEUCINE METABOLISM

R114a : Isoleucine_A + alpha-ketoglutarate_A → KMV_A + Glutamate_A

R115a : KMV_A + NAD_AM → Methylbutyryl-CoA_A + CO2_A + NADH_AM

R116a : Methylbutyryl-CoA_A + FAD_AM + NAD_AM → FADH2_AM + NADH_AM + Propionyl-CoA_A + Acetyl-CoA_A

R117c : KMV_A → KMV_N

R118n : KMV_N + Glutamate_N → Isoleucine_N + alpha-ketoglutarate_N

R119c : Isoleucine_N → Isoleucine_A

LYSINE METABOLISM

R120n : Lysine_N + alpha-ketoglutarate_N + NADPH_N → Saccharopine_N + NADP_N

R121n : Saccharopine_N + NAD_N → Glutamate_N + NADH_N + 2-aminoadipate semialdehyde_N

R122n : 2-aminoadipate semialdehyde_N + alpha-ketoglutarate_N + 1 NAD_N → alpha-ketoadipate_N + Glutamate_N + 1 NADH_N

R123n : alpha-ketoadipate_N + 2 NAD_NM + FAD_NM → Acetoacetyl-CoA_N + 2 NADH_NM + FADH2_NM + 2 CO2_N

R124n : Acetoacetyl-CoA_N → 2 Acetyl-CoA_N

PHENYLALANINE-TYROSINE METABOLISM

R125n : Phenylalanine_N + O2_N + NADPH_N → Tyrosine_N + NADP_N

R126n : Tyrosine_N + O2_N → L-DOPA_N

R127n : L-DOPA_N → Dopamine_N + CO2_N

R128n : Dopamine_N + O2_N → Norepinephrine_N

R129n : Norepinephrine_N → Epinephrine_N

R130c : Dopamine_N → Dopamine_A

R131a : Dopamine_A → Norepinephrine_A

TRYPTOPHAN METABOLISM

R132n : Tryptophan_N + O2_N → 5- hydroxytryptophan_N

R133n : 5- hydroxytryptophan_N → Seratonin_N + CO2_N

R134n : Seratonin_N → Melatonin_N

ACETYLCHOLINE METABOLISM

R135n : Choline_N + Acetyl-CoA_N ↔ Acetylcholine_N

CHOLESTEROL SYNTHESIS (Astrocyte Only)

R136a: 18 Acetyl-CoA_A + 18 ATP_A + 11 O2_A + 29 NADPH_A + 2 NAD_A → 9 CO2_A + Cholesterol_A + 2 NADH_A + 18 ADP_A + 29 NADP_A
R137c: Cholesterol_A → Cholesterol_N

FATTY ACID SYNTHESIS

R138a : 8 Acetyl-CoA_A + 7 ATP_A + 14 NADPH_A → Palmitate_A + 7 ADP_A + 14 NADP_A
R139a : 9 Acetyl-CoA_A + 8 ATP_A + 16 NADPH_A → Stearate_A + 8 ADP_A + 16 NADP_A
R140a : 9 Acetyl-CoA_A + 8 ATP_A + 15 NADPH_A → Oleate_A + 8 ADP_A + 15 NADP_A
R141a : Linoleate_A + 2 O2_A + 2 NADH_A + ATP_A + CO2_A + Acetyl-CoA_A → Arachidonate_A + ADP_A + 2 NAD_A
R142a : Linolenate_A + 3 O2_A + 3 NADH_A + 2 ATP_A + 2 CO2_A + 2 Acetyl-CoA_A → Decosahexenoate_A + 2 ADP_A + 3 NAD_A

R143n : 8 Acetyl-CoA_N + 7 ATP_N + 14 NADPH_N → Palmitate_N + 7 ADP_N + 14 NADP_N
R144n : 9 Acetyl-CoA_N + 8 ATP_N + 16 NADPH_N → Stearate_N + 8 ADP_N + 16 NADP_N
R145n : 9 Acetyl-CoA_N + 8 ATP_N + 15 NADPH_N → Oleate_N + 8 ADP_N + 15 NADP_N
R146c : Arachidonate_A → Arachidonate_N
R147c : Decosahexenoate_A → Decosahexenoate_N

LIPID SYNTHESIS (Astrocytes & Neurons)

R148a : 0.270 Palmitate_A + 0.276 Stearate_A + 0.249 Oleate_A + 0.108 Arachidonate_A + 0.094 Decosahexenoate_A → 1 FattyAcid_A
R149n : 0.270 Palmitate_N + 0.276 Stearate_N + 0.249 Oleate_N + 0.108 Arachidonate_N + 0.094 Decosahexenoate_N → 1 FattyAcid_N

R150a : Dihydroxyacetone-phosphate_A + NADH_A → Glycerol-3-phosphate_A + NAD_A

R151a : Glycerol-3-phosphate_A + FAD_AM → Dihydroxyacetone-phosphate_A + FADH2_AM

R152a : Glycerol-3-phosphate_A + 2 FattyAcid_A → Phosphatidate_A

R153a : Phosphatidate_A + 3 ATP_A + Ethanolamine_A → 3 ADP_A + Phosphatidyl-ethanolamine_A

R154a : Phosphatidyl-ethanolamine_A → Phosphatidyl-choline_A

R155a : Choline_A + 3 ATP_A + Phosphatidate_A → Phosphatidyl-choline_A + 3 ADP_A

R156a : Phosphatidyl-ethanolamine_A + Serine_A → Ethanolamine_A + Phosphatidyl-serine_A

R157a : Phosphatidyl-serine_A → Phosphatidyl-ethanolamine_A + CO2_A

R158n : Dihydroxyacetone-phosphate_N + FADH2_NM → Glycerol-3-phosphate_N + FAD_NM

R159n : Glycerol-3-phosphate_N + 2 FattyAcid_N → Phosphatidate_N

R160n : Phosphatidate_N + 3 ATP_N + Ethanolamine_N → 3 ADP_N + Phosphatidyl-ethanolamine_N

R161n : Phosphatidyl-ethanolamine_N → Phosphatidyl-choline_N

R162n : Choline_N + 3 ATP_N + Phosphatidate_N → Phosphatidyl-choline_N + 3 ADP_N

R163n : Phosphatidyl-ethanolamine_N + Serine_N → Ethanolamine_N + Phosphatidyl-serine_N

R164n : Phosphatidyl-serine_N → Phosphatidyl-ethanolamine_N + CO2_N

R165a : 0.52 Cholesterol_A + 0.24 Phosphatidyl-ethanolamine_A + 0.23 Phosphatidyl-choline_A + 0.09 Phosphatidyl-serine_A → Lipid_A

R166n : 0.52 Cholesterol_N + 0.24 Phosphatidyl-ethanolamine_N + 0.23 Phosphatidyl-choline_N + 0.09 Phosphatidyl-serine_N → Lipid_N

REACTIVE oXYGEN SPECIES (ROS) PATHWAY

R167a : Cystine_A + NADH_A → 2 Cysteine_A + NAD_A

R168a : Cysteine_A + Glutamate_A + Glycine_A + 2 ATP_A → ReducedGlutathione_A + 2 ADP_A

R169a : O2_A → H2O2_A

R170a : 2 ReducedGlutathione_A + H2O2_A → OxidizedGlutathione_A

R171a : OxidizedGlutathione_A + NADPH_A → 2 ReducedGlutathione_A + NADP_A

R172a : OxidizedGlutathione_A + NADPH_AM → 2 ReducedGlutathione_A + NADP_AM

R173a : H2O2_A → O2_A

R174a : ReducedGlutathione_A + Glutamine_A → Glutamate_A + NH3_A + CysteinylGlycine_A

R175c : CysteinylGlycine_A → CysteinylGlycine_N

R176n : CysteinylGlycine_N → Cysteine_N + Glycine_N

R177n : Cysteine_N + Glutamate_N + Glycine_N + 2 ATP_N → ReducedGlutathione_N + 2 ADP_N

R178n : O2_N → H2O2_N

R179n : 2 ReducedGlutathione_N + H2O2_N → OxidizedGlutathione_N

R180n : OxidizedGlutathione_N + NADPH_N → 2 ReducedGlutathione_N + NADP_N

R181n : OxidizedGlutathione_N + NADPH_NM → 2 ReducedGlutathione_N + NADP_NM

R182n: H2O2_N → O2_N

GLYCOGEN DEGRADATION METABOLISM (active in stressed conditions)

R183a : Glycogen → Glucose-1-phosphate_A

R184a : Glucose-1-phosphate_A → Glucose-6-phosphate_A

EXCHANGE REACTIONS

LACatr : Lactate_A → Lactate_AEX

LACntr : Lactate_N → Lactate_NEX

CO2atr : CO2_A → CO2_AEX

CO2ntr : CO2_N → CO2_NEX

GLNatr : Glutamine_A → Glutamine_AEX

DOPntr : Dopamine_N → Dopamine_NEX

NOREPntr: Norepinephrine_N → Norepinephrine_NEX

EPntr : Epinephrine_N → Epinephrine_NEX

NOREPatr: Norepinephrine_A → Norepinephrine_AEX

SERTntr : Seratonin_N → Seratonin_NEX

MELTntr : Melatonin_N → Melatonin_NEX

CHLNntr : Acetylcholine_N → Acetylcholine_NEX

LPDntr : Lipid_N → Lipid_NEX

LPDatr : Lipid_A → Lipid_AEX

LNLTatr : Linoleate_AEX → Linoleate_A

LNLNTatr : Linolenate_AEX → Linolenate_A

CHLntr : Choline_NEX → Choline_N

CHLatr : Choline_AEX → Choline_A

CYSatr : Cystine_AEX → Cystine_A

GSHntr : ReducedGlutathione_N → ReducedGlutathione_NEX

GLCatr : GLC_AEX → Glucose_A

GLCntr : GLC_NEX → Glucose_N

O2atr : O2_AEX → O2_A

O2ntr : O2_NEX → O2_N

LEUatr : LEU_AEX → Leucine_A

ILEatr : ILE_AEX → Isoleucine_A

VALatr : VAL_AEX → Valine_A

TYRntr : TYR_NEX → Tyrosine_N

TRPntr : TRP_NEX → Tryptophan_N

LYSntr : LYS_NEX → Lysine_N

PHEntr : PHE_NEX → Phenylalanine_N

NH3atr : NH3_AEX → NH3_A
