# Supplementary material for: Reconstruction and flux analysis of coupling between metabolic pathways of astrocytes and neurons: application to cerebral hypoxia
Source: Theor Biol Med Model. 2007 Dec 10;4:48. doi: 10.1186/1742-4682-4-48 (PMC2246127; doi:10.1186/1742-4682-4-48)
Supplement: Additional file 2 — Supplementary Table 1 – Metabolic differences in two cell types reflected in our model. [file 1742-4682-4-48-S2.pdf]

Supplementary Table 1.a: Metabolic differences in two cell types reflected in our model for pathway (internal) reactions

Reaction/ Enzyme	Reaction Number in astrocytes	Reaction Number in neurons	Coupling/ Differentiation
Lactate dehydrogenase	r_{11}	-	only active in astrocytes
pyruvate carboxylation	r_{12}	-	only active in astrocytes
cytosolic malate dehydrogenation	r_{32}	r_{68}	The one in neurons is in reverse direction of the mitochondrial reaction, thereby mimicking malate-aspartate shuttle active in neurons
malic enzyme	r_{33}	r_{69}	cytosolic in astrocytes, mitochondrial in neurons
glutamine synthetase	r_{77}	-	only active in astrocytes
glutaminase	-	r_{79}	only active in neurons
glutamate decarboxylase	-	r_{80}	only active in neurons
3-phosphoglycerate dehydrogenase	r_{92}	-	only active in astrocytes
serine dehydratase	r_{93}	-	only active in astrocytes
glycine cleavage system	r_{97}	-	only active in astrocytes
branched-chain keto-acid dehydrogenase	r_{100}, r_{108}, r_{115}	-	only active in astrocytes
branched-chain acyl-coa dehydrogenase	r_{101}, r_{109}, r_{116}	-	only active in astrocytes
lysine catabolism	-	r_{120}-r_{123}	only active in neurons
phenylalanine catabolism	-	r_{125}-r_{129}	only active in neurons
tyrosine catabolism	-	r_{132}-r_{134}	only active in neurons
acetylcholine metabolism	-	r_{135}	only active in neurons
cholesterol synthesis	r_{136}	-	only active in astrocytes. Astrocytes supply tnecessary cholesterol to neurons.
Arachidonate and Decosahexenoate synthesis	r_{141}, r_{142}	-	only active in astrocytes. Astrocytes supply these fatty acids to neurons.
cytosolic glycerol 3-phosphate dehydrogenase	r_{150}	-	only active in astrocytes
mitochondrial glycerol 3-phosphate dehydrogenase	r_{151}	r_{158}	The one in astrocytes is in reverse direction of the cytosolic reaction, thereby mimicking glycerol 3-phosphate shuttle active in astrocytes
cystine reductase	r_{167}	-	only active in astrocytes
glycogen phosphorylase	r_{183} , r_{184}		only active in astrocytes in stress conditions

Supplementary Table 1.b: Metabolic differences in two cell types reflected in our model for exchange reactions

Lactate release	only active in astrocytes
glutamine release	only active in astrocytes
Dopamine release	only active in neurons
epinephrine release	only active in neurons
serotonin release	only active in neurons
melatonin release	only active in neurons
acetylcholine release	only active in neurons
linoleate uptake	only active in astrocytes
linolenate uptake	only active in astrocytes
cystine uptake	only active in astrocytes
leucine uptake	only active in astrocytes
isoleucine uptake	only active in astrocytes
valine uptake	only active in astrocytes
tyrosine uptake	only active in neurons
lysine uptake	only active in neurons
phenylalanine uptake	only active in neurons
ammonia uptake	only active in astrocytes
Glycogen breakdown	only active in astrocytes
