# Supplementary material for: Reconstruction and flux analysis of coupling between metabolic pathways of astrocytes and neurons: application to cerebral hypoxia
Source: Theor Biol Med Model. 2007 Dec 10;4:48. doi: 10.1186/1742-4682-4-48 (PMC2246127; doi:10.1186/1742-4682-4-48)
Supplement: Additional file 3 — Employed objective functions with corresponding reasonings. [file 1742-4682-4-48-S3.pdf]

**Supplementary Table 2**: Employed objective functions with corresponding reasonings

| **objective function** | **Reasoning** | **Literature application** | **Deficits in the Simulation Results** |
|---|---|---|---|
| minimization of squared sum of total fluxes (Euclidean norm) | Cells try to minimize the load on their enzymes, trying to perform its duties by spending its sources (enzymes) in minimum amount. | (Bonarius *et al.*, 1996 ; Holzhutter, 2004) | - zero Lactate release flux<br>- zero flux for Glutamate transfer from neurons to astrocytes ($r_{76}$) |
| minimization of redox potential | This can be interpreted as maximization of energy efficiency. By minimizing the oxidation, energy is conserved. | (Knorr *et al.*, 2007 ; Savinell & Palsson, 1992) | - low Lactate release flux (1% of glucose)<br>- zero flux for glutamate/ glutamine/ GABA cycles ($r_{76}$,$r_{79}$,$r_{82}$)<br>- zero BCAA shuttle fluxes |
| minimization of ATP production | This implies efficient use of energy. Cell is forced to perform its functions with minimal energy need, leading ATP conservation. | (Knorr *et al.*, 2007) | - zero Lactate release flux<br>- zero flux for Glutamate transfer from neurons to astrocytes<br>- inactive astrocytic PPP flux ($r_{14}$) |
| maximization of ATP production | Production of energy is aimed for other processes not considered in the model | (Beasley & Planes, 2007 ; Ramakrishna *et al.*, 2001) | - low Lactate release flux (1.5 % of glucose)<br>- inactive neuronal PPP flux ($r_{51}$)<br>- zero flux for glutamate/ glutamine/ GABA cycles ($r_{76}$,$r_{79}$,$r_{82}$) |
| Maximization of transfer fluxes from astrocytes to neurons | Astrocytes are support cells for neurons. This objective ensures highest support by astrocytes to neurons. | intuition | - zero Lactate release flux<br>- zero flux for glutamate/ glutamine/ GABA cycles ($r_{76}$,$r_{79}$,$r_{82}$)<br>- very high neuronal oxidative metabolism (7-fold higher than astrocytic one) |

| | | | |
|---|---|---|---|
| ATP maximization for neurons, ATP minimization for astrocytes | Each cell type is assumed to have different, but interrelated, objectives. Neurons are known to have high oxidative metabolism whereas this is not the case for astrocytes. | intuition | - zero Lactate release flux<br>- zero flux for glutamate/ glutamine/ GABA cycles ($r_{76}$,$r_{79}$,$r_{82}$)<br>- inactive neuronal PPP flux ($r_{51}$) |
| Maximization of NADPH producing reactions | Cells need NADPH for protection from oxidative stress and for lipid synthesis | (Fell & Small, 1986) | - zero Lactate release flux<br>- zero flux for Glutamate transfer from neurons to astrocytes<br>- very low flux for return part of the glutamate-glutamine cycle ($r_{79}$) and GABA cycle |
| maximization of malate shuttle in neurons | Support of oxidative metabolism of neurons is aimed by transferring optimum amount of NADH from cytosol to mitochondria. | intuition | - zero Lactate release flux<br>- zero flux for Glutamate transfer from neurons to astrocytes<br>- low flux for return part of the glutamate-glutamine cycle (r79) and GABA cycle<br>- zero flux of BCAA cycling |
| maximization of glutamine transfer from astrocytes to neurons | Astrocytes are considered as glutamate producers for neurons | intuition | - inactive astrocytic PPP flux ($r_{14}$) |
| **Maximization of glutamate-glutamine-GABA exchange between astrocytes and neurons** | **glutamate, glutamine and GABA constitute the most important trafficking between astrocytes and neurons** | **intuition** | |

## References

Beasley JE, Planes FJ (2007) Recovering metabolic pathways via optimization.. Bioinformatics 23: 92-98.

Bonarius HPJ, Hatzimanikatis V, Meesters KPH, de Gooijer CD, Schmid G et al. (1996) Metabolic flux analysis of hybridoma cells in different culture media using mass balances. Biotechnol Bioeng 50: 299-318.

Fell DA, Small JR (1986) Fat synthesis in adipose tissue. an examination of stoichiometric constraints. Biochem J 238: 781-786.

Holzhutter H (2004) The principle of flux minimization and its application to estimate stationary fluxes in metabolic networks. Eur J Biochem 271: 2905-2922.

Knorr AL, Jain R, Srivastava R (2007) Bayesian-based selection of metabolic objective functions.. Bioinformatics 23: 351-357.

Ramakrishna R, Edwards JS, McCulloch A, Palsson BO (2001) Flux-balance analysis of mitochondrial energy metabolism: consequences of systemic stoichiometric constraints.. Am J Physiol Regul Integr Comp Physiol 280: R695-704.

Savinell JM, Palsson BO (1992) Network analysis of intermediary metabolism using linear optimization. i. development of mathematical formalism.. J Theor Biol 154: 421-454.
